# Overexpression of *Enterococcus faecalis elr* operon protects from phagocytosis

**DOI:** 10.1186/s12866-015-0448-y

**Published:** 2015-05-25

**Authors:** Naima G. Cortes-Perez, Romain Dumoulin, Stéphane Gaubert, Caroline Lacoux, Francesca Bugli, Rebeca Martin, Sophie Chat, Kevin Piquand, Thierry Meylheuc, Philippe Langella, Maurizio Sanguinetti, Brunella Posteraro, Lionel Rigottier-Gois, Pascale Serror

**Affiliations:** INRA, UMR1319 Micalis, Jouy-en-Josas, France; AgroParisTech, UMR1319 Micalis, Jouy-en-Josas, France; Institute of Microbiology, Università Cattolica del Sacro Cuore, Rome, Italy; Institute of Public Health (Section of Hygiene), Università Cattolica del Sacro Cuore, Rome, Italy; Current address: INRA, Unité d’Immuno-Allergie Alimentaire, iBiTecS/SPI, Gif-sur-Yvette, France

**Keywords:** *Enterococcus faecalis*, Macrophage, *elrA*, *elr* operon

## Abstract

**Background:**

Mechanisms underlying the transition from commensalism to virulence in *Enterococcus faecalis* are not fully understood. We previously identified the enterococcal leucine-rich protein A (ElrA) as a virulence factor of *E. faecalis*. The *elrA* gene is part of an operon that comprises four other ORFs encoding putative surface proteins of unknown function.

**Results:**

In this work, we compared the susceptibility to phagocytosis of three *E. faecalis* strains, including a wild-type (WT), a Δ*elrA* strain, and a strain overexpressing the whole *elr* operon in order to understand the role of this operon in *E. faecalis* virulence. While both WT and Δ*elrA* strains were efficiently phagocytized by RAW 264.7 mouse macrophages, the *elr* operon-overexpressing strain showed a decreased capability to be internalized by the phagocytic cells. Consistently, the strain overexpressing *elr* operon was less adherent to macrophages than the WT strain, suggesting that overexpression of the *elr* operon could confer *E. faecalis* with additional anti-adhesion properties. In addition, increased virulence of the *elr* operon-overexpressing strain was shown in a mouse peritonitis model.

**Conclusions:**

Altogether, our results indicate that overexpression of the *elr* operon facilitates the *E. faecalis* escape from host immune defenses.

**Electronic supplementary material:**

The online version of this article (doi:10.1186/s12866-015-0448-y) contains supplementary material, which is available to authorized users.

## Background

As a natural inhabitant of the oral cavity, gastrointestinal tract, and female vaginal tract in humans, *Enterococcus faecalis* is normally considered a nonpathogenic microorganism. However, it is a common opportunistic pathogen in immunocompromised patients, causing nosocomial infections. While our current understanding of the mechanisms that lead to the lifestyle shift from commensalism to virulence in enterococci remains an emerging area of research, the pathogenesis of *E. faecalis* is clearly nonetheless a complex multifactorial process that currently remains poorly understood. In this regard, we have previously identified the enterococcal leucine-rich protein A (ElrA), a protein that possesses a leucine-rich repeat (LRR) domain and a carboxy-terminal WxL domain, which promotes non-covalent association to the bacterial surface [1]. ElrA is encoded by the *elr* operon , which encodes two other WxL surface proteins, a small LPXTG-motif protein and a putative transmembrane protein proposed to form cell surface complexes [1–4]. Expression of the *elr* operon is under the control of the positive regulator *elrR* [4, 5]. The *elrA* gene is poorly expressed *in vitro*, but it can be induced by complex biological milieu such as serum or urine, which suggests the tightly regulated control of *elrA* expression in response to *in vivo* signals [5, 6]. Previously, we showed that inactivation of the *elrA* gene resulted in significantly reduced virulence in a mouse model of peritonitis [4]. We also observed reduced secretion of interleukin-6 (IL-6, a pro-inflammatory cytokine) upon *in vivo* infection with the Δ*elrA* mutant strain and we hypothesized that ElrA may be involved in this modulation, by stimulating host immune cells to counteract *E. faecalis* infection [4].

Macrophages are potent antigen presenting cells that play a key role in initiating an immune response against invading bacteria. In turn, some pathogens have evolved strategies in order to circumvent macrophage functions [7]. Previous studies have shown that *E. faecalis* can survive in peritoneal macrophages better than other non-pathogenic bacteria [8, 9]. In addition, it possesses mechanisms permitting escape from murine or human macrophages [8, 10, 11]. *E. faecalis* cell wall glycopolymers play a key role in the resistance to phagocytosis. In particular, capsular polysaccharide serotypes C and D contribute to complement evasion [12, 13] and rhamnopolysaccharide Epa protects from phagocytic killing [13, 14], most likely by preventing uptake by macrophages as we recently showed in zebrafish model [15].

In the present study, we sought to evaluate whether the expression of *elrA* alone or that of the entire *elr* operon most influences the capability of *E. faecalis* to be phagocytized by the RAW 264.7 mouse macrophages *in vitro*. To circumvent the aforementioned low level of *elrA* expression *in vitro*, a genetically modified *E. faecalis* strain harboring a constitutive promoter upstream of the *elr* operon (P^+^-*elrA*-*E*) was constructed. The ability of this *elr*-overexpressing strain to be internalized was compared with a wild-type strain of *E. faecalis*, and with different isogenic-*elr* mutant strains, obtained by genetic manipulation of the *E. faecalis* P^+^-*elrA*-*E* strain.

## Results and discussion

### Production of ElrA requires other gene(s) of the *elr* operon

**Fig. 1:**
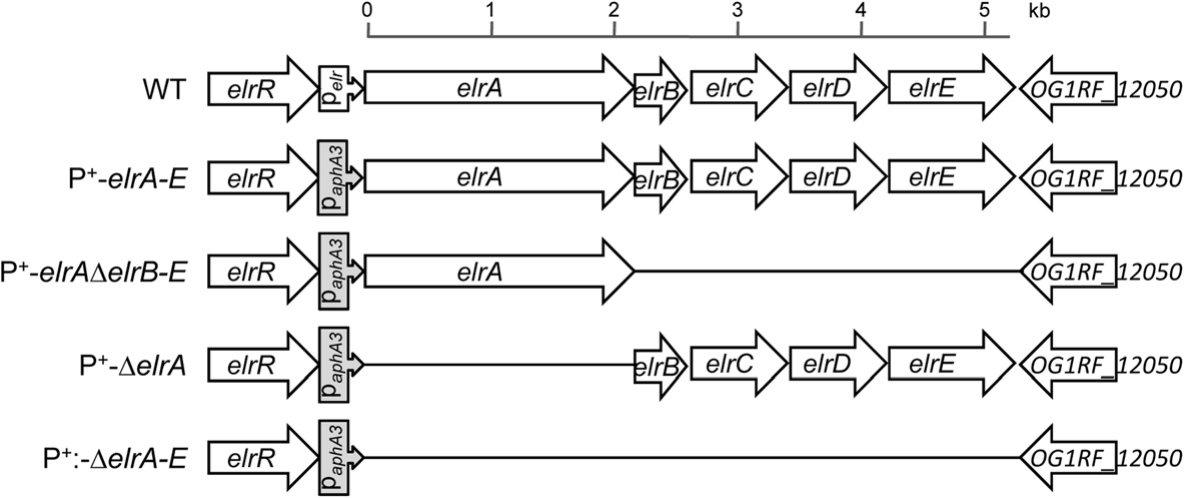
Schematic representation of the *elrA* operon in strains used. In *E. faecalis* WT *elrA* is followed by four genes encoding proteins of unknown function (*OG1RF_12054* or *elrB* to *OG1RF_12051* or *elrE*). The gene products are all predicted secreted proteins with an amino-terminal signal peptide. ElrA, ElrC and ElrD display a C-terminal WxL domain. ElrB possess a carboxy-terminal LPxTG anchor. ElrE belongs to the DUF916 family protein and has a predicted C-terminal transmembrane anchor. In P^+^-*elrA-E* the natural *elrA* promoter was replaced by the constitutive promoter of the kanamycin resistance gene (P_*aphA3*_). The P^+^-*elrA-E* strain was used as recipient for all mutant constructions

As previously discussed, *E. faecalis* ElrA protein is poorly expressed *in vitro*, but induced *in vivo* and is particularly important for *E. faecalis* virulence [4, 5]. Moreover, this protein could not be detected by Western blot experiments in total protein extracts prepared from *E. faecalis* wild-type strain OG1RF (WT) [4, 5]. Located immediately downstream of *elrA* in the five-gene *elr* operon (see Materials and Methods and Fig. 1), there is a gene encoding a small protein with an LPXTG anchor motif (ElrB), followed by two further proteins each possessing a carboxy-terminal WxL anchor motif (ElrC and ElrD), and finally a putative transmembrane protein (ElrE), a member of the DUF916 protein family [4]. A BLAST (Basic Local Alignment Search Tool) analysis performed on the sequence of *elrABCDE* using the NCBI non-redundant protein sequence (nr) database revealed novel orthologs for the five proteins (ElrA-ElrE). The corresponding best matches for ElrA were observed with the hypothetical protein WP_022792020.1 of *Weissella halotolerans* (34 % identity and 49 % homology between residues 85 to 718 of ElrA and residues 8 to 680 of WP_022792020.1) and the hypothetical protein UC3_01347 of *Enterococcus phoeniculicola* (36 % identity and 54 % homology between residues 1 to 471 of ElrA and residues 1 to 467 of UC3_01347), followed by InlA of *L. monocytogenes* as initially reported [4]. Orthologs of proteins ElrB to ElrE were detected in various species with the respective best matches for *Enterococcus pallens* (ElrB), *Enterococcus phoeniculicola* (ElrC), *Lactococcus garvieae* and *Enterococcus avium* (ElrD), *Carnobacterium divergens* and *Carnobacterium maltaromaticum* (ElrE). Proteins possessing WxL domains, cognate putative transmembrane and LPXTG proteins have been proposed to form multicomponent complexes on the bacterial surface [2]. This hypothesis is supported by the recent work of Galloway-Pena *et al.* who showed interaction between *E. faecium* locus A-encoded WxL proteins and the cognate transmembrane protein *in vitro* [3]. In this context, the organization of the *elr* operon suggests that the four proteins (ElrB-ElrE) have a function related to ElrA. To address the role of ElrA *in vitro* while maintaining Elr protein stoichiometry, we engineered an *E. faecalis* strain overexpressing the whole *elr* operon. This strain, *E. faecalis* P^+^-*elrA-E*, was generated by replacement of the *elr* operon promoter region by the *E. faecalis* P_*aphA3*_ constitutive promoter (P^+^ strain, see Material and Methods) [4, 16] (Fig. 1). Subsequently, to explore the role of ElrB-ElrE in ElrA function, the P^+^-*elrA*-*E* strain was used to generate: i) a strain expressing the *elr* operon but without *elrA*: *E. faecalis* P^+^-Δ*elrA*, ii) a strain overexpressing only *elrA*: *E. faecalis* P^+^-*elrA-*Δ*elrB*-*E* strain, and iii) a strain where the entire *elr* operon was inactivated: *E. faecalis* P^+^-Δ*elrA*-*E* (Fig. 1). Growth of the three strains was comparable (data not shown), indicating that neither expression of *elr* operon nor parts of it impacted bacterial growth under the conditions tested.

**Fig. 2:**
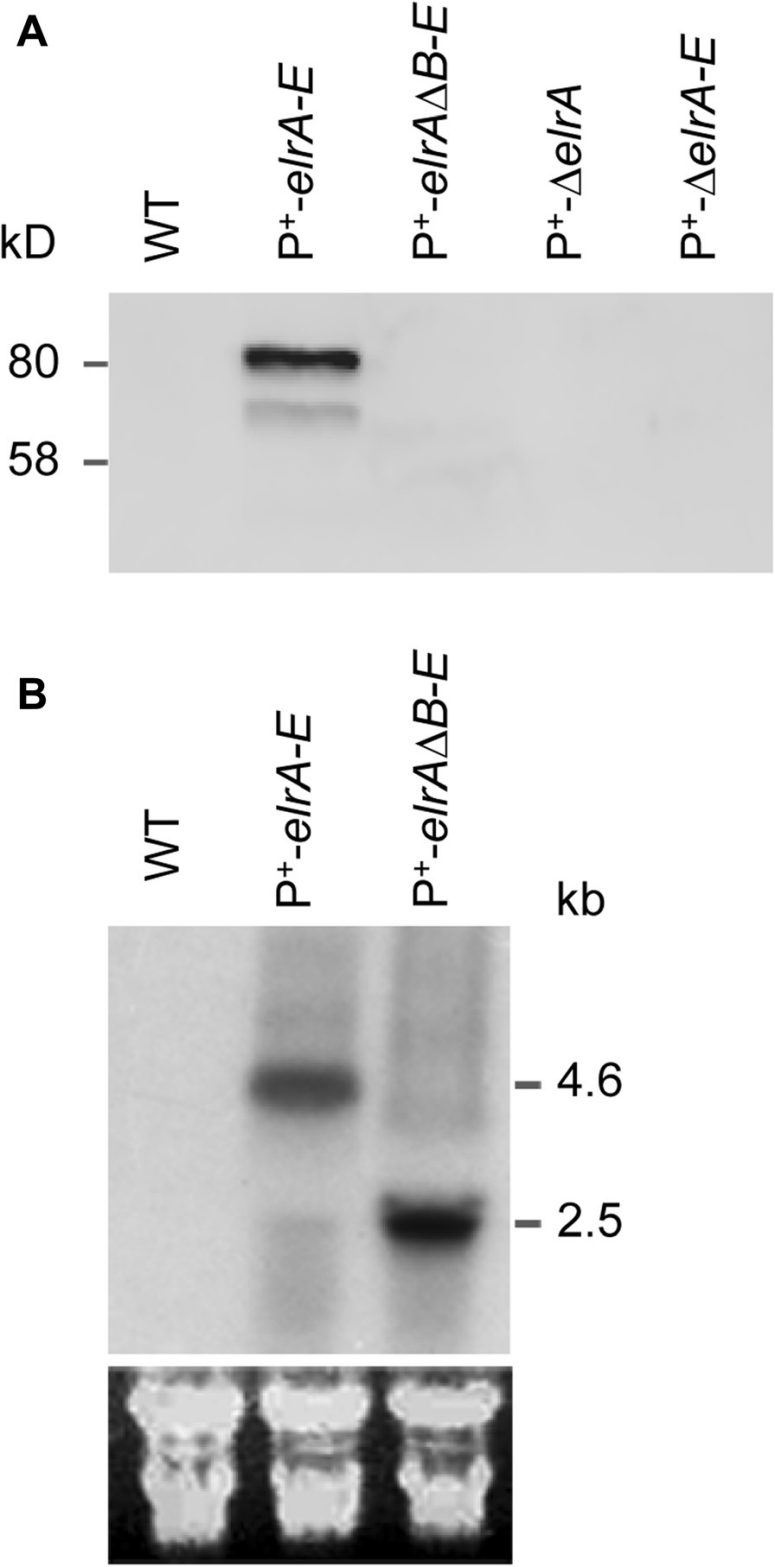
Detection of ElrA protein and *elr* transcript. **a**) Western blot analysis of total protein extracts from WT and mutant strains of *E. faecalis*, that was performed using a 12 % SDS-PAGE and polyclonal rat anti-ElrA antibodies, is shown. Band at ~80kD corresponds to the predicted size of ElrA, whereas the additional band represents a degradation product. **b**) Northern blot analysis of *elr* operon performed with ~40 μg of total RNA which was extracted from exponentially growing cells. Names of strains analyzed are indicated at the top of each lane. Probes used were *elrA-*specific oligonucleotide probes. The estimated length of transcripts that agrees with their predicted sizes is shown on the right. Below, ribosomal RNAs were used as loading controls

We first tested ElrA production in each of the different strains by Western blot analysis. As expected, ElrA was not detected in protein extracts prepared from WT, P^+^-Δ*elrA,* or P^+^-Δ*elrA*-*E* culture. Strikingly, protein extracts prepared from P^+^-*elrA*-*E* strain revealed a band of the expected size for ElrA (80-kDa, Fig. 2A), confirming that the endogenous P_*elrA*_ promoter is inactive *in vitro*, and that its replacement by a constitutive promoter allows expression of *elrA in vitro*. No ElrA was detected in protein extracts prepared from the P^+^-*elrA-*Δ*elrB-E* strain (Fig. 2A), suggesting an important role for at least one of the four other proteins present in the *elr* operon in either the production or stability of ElrA. To corroborate this hypothesis and study *elrA* transcription in the P^+^-*elrA*-Δ*elrB*-*E* strain, *elrA* transcripts were analyzed by Northern blotting hybridization using total RNA prepared from the WT, P^+^-*elrA*-*E* and P^+^-*elrA-*Δ*elrB*-*E* strains. As expected, *elrA* transcript was not detected in the WT strain by Northern blotting under laboratory growth conditions, confirming our previous results [4, 5]. Similar analyses in respect of the P^+^-*elrA-E* and P^+^-*elrA-*Δ*elrB-E* overexpression strains resulted in strong hybridization signals corresponding to transcripts, of sizes of ~5 and 2.4 kb, corresponding to the predicted full-length, and the *elrBCDE*-deleted *elr* operon transcripts, respectively (Fig. 2B). Detection of *elrA* transcripts in P^+^-*elrA-*Δ*elrB-E* strain strongly indicates a post-transcriptional control of ElrA expression, confirming the important role of at least one of the four other *elr* operon genes for either ElrA production or stabilization.

### Overexpression of *elr* operon impairs phagocytosis

**Fig. 3:**
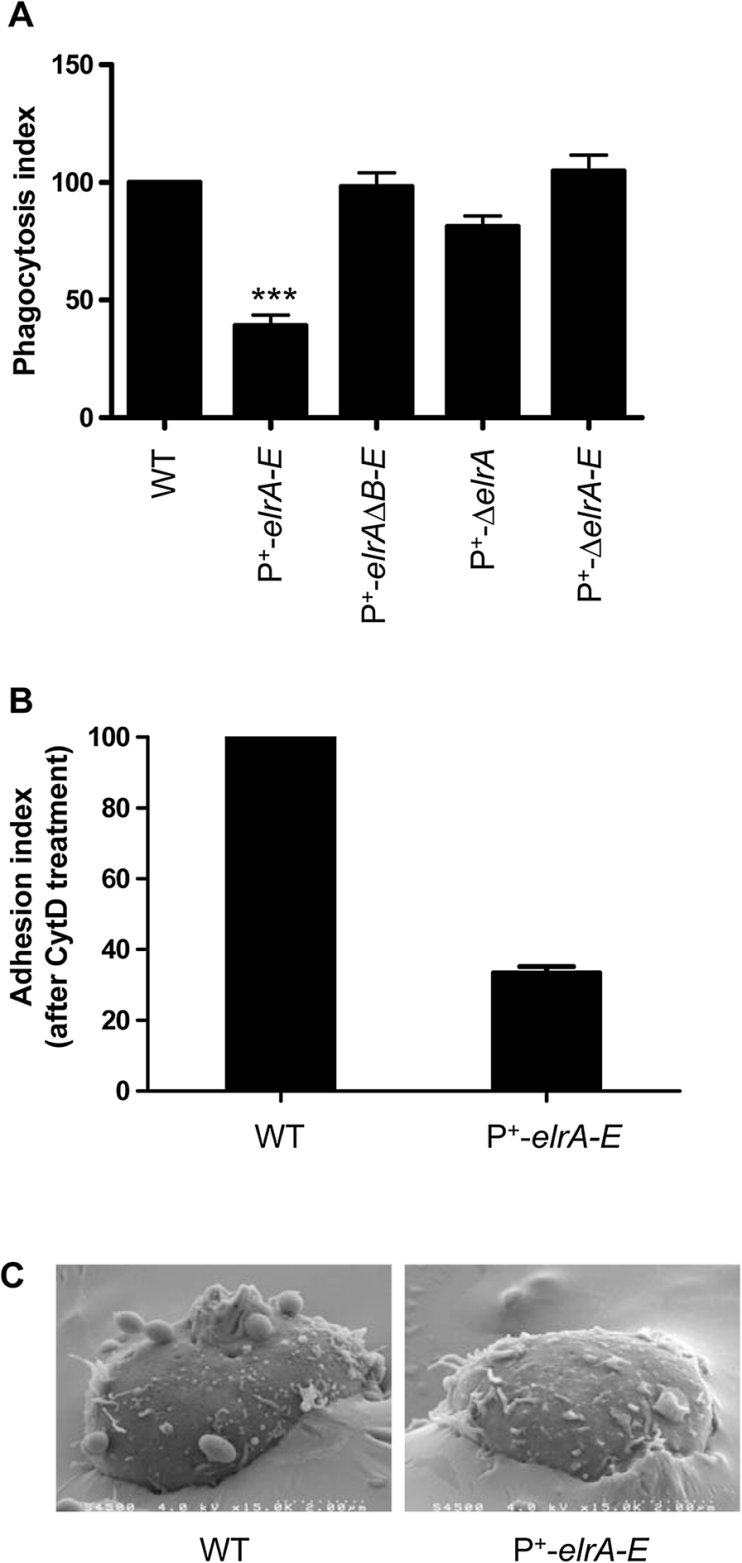
Phagocytosis of isogenic strains overexpressing full-length or partially deleted *elr* operon by RAW macrophages. **a**) For all *E. faecalis* strains tested, the phagocytosis index (PI) was calculated as average ± SEM from three independent experiments. Statistical significance was measured by ANOVA and Dunnett's multiple comparison test, ***P< 0.001. **b**) Adhesion index (AI) of *E. faecalis* strains after treatment with cytochalasin D. AI was calculated as follows: AI = % of GFP-labeled macrophages after infection with the mutant strain X 100/% of GFP-labeled macrophages after infection with the WT strain. Shown is the mean ± SEM from two independent experiments performed in duplicate. **c**) Scanning electronic microscopy (SEM) showing *E. faecalis* adhesion. Micrographs of macrophages infected for 30 min with *E. faecalis* strains observed by electron scanning microscopy. The micrographs are representative of two independent experiments

We then explored the effect of *elr* operon overexpression on the *E. faecalis* interaction with macrophages by monitoring phagocytosis of GFP-labeled *E. faecalis* strains by RAW cells using flow cytometry analysis. Firstly, we evaluated the phagocytosis dynamics of the *E. faecalis* WT strain at different multiplicities of infection (MOI, data not show) and decided to use a MOI of 1:100 in which approximately 58 % ±11 (mean ± SEM after three independent experiments each one in triplicate) of macrophages were GFP positive after 30 min of interaction. This value was used as reference (nominal 100 %) in order to estimate the phagocytosis index (PI) of the different *E. faecalis* mutant strains (see Materials and Methods). Operon inactivation (P^+^-Δ*elrA-E*) did not affect bacterial uptake when compared to the WT strain (105 ± 6.7 %, Fig. 3A). In contrast, a significant reduction of phagocytosis was observed with P^+^-*elrA-E* strain (PI = 39 % ± 4.4; *P* <0.0001, Fig. 3A). Although constitutive expression of the four other operon proteins (P^+^-Δ*elrA* strain) appeared to reduce phagocytosis (PI = 81.3 % ± 4.4), this reduction was not statistically significant (Fig. 3A). As expected, given that the P^+^-*elrA-*Δ*elrB-E* strain does not appear to produce ElrA (Western blot results, Fig. 2A), no difference in phagocytosis was observed when this strain was compared to the WT strain (PI = 98.2 % ± 5.8) (Fig. 3A). Lower levels of uptake by phagocytosis of the strain P^+^-*elrA-E* compared to the WT was confirmed by double-labeling fluorescence microscopy analysis (data not shown). These results support a link between the expression of *elr* operon and the uptake of *E. faecalis*.

### Overexpression of *elr* operon modifies bacterial adhesion

Phagocytosis is initiated with the recognition of ligands on bacterial cell surfaces by receptors including scavenger receptors, glucan receptors, and integrins present on the membrane of macrophages, which leads to bacteria engulfment via an actin-dependent mechanism. To test whether the impairment of phagocytosis seen for the P^+^-*elrA-E* strain correlated with reduced adhesion of the bacterium to macrophage cells, we used cytochalasin D (CytD), which inhibits phagocytosis, but does not prevent the initial step of bacterial adhesion [17, 18]. Macrophages were therefore infected (as described above for the phagocytosis test) with either WT or P^+^-*elrA-E* strains in the presence or absence of CytD, and the percentages of GFP^+^ macrophages were measured by flow cytometry analysis (uninfected macrophages were used as negative control). Comparison of forward scatter (FSC) and side scatter (SSC) values from uninfected cells (CytD treated or untreated) confirmed that macrophages were not altered by CytD (Additional file 1: Figure S1). As shown in Fig. 3B, P^+^-*elrA-E* strain was 60 % less adherent to macrophages than the WT strain. These results are in agreement with scanning microscopy observations of infected macrophages, that showed a sharp contrast between adhesion of WT and P^+^-*elrA-E* strains (Fig. 3C).

Because proteins encoded by the *elr* operon demonstrate characteristics of surface proteins (WxL, and LPXTG motifs) and could form a surface complex, we hypothesized that overexpression of *elr* operon could result in the formation of surface structures, which in turn resulted in the inhibition of phagocytosis as observed *in vitro*. Analyses of bacterial strains using transmission electron microscopy (thin sections and negative staining) and scanning electron microscopy revealed no differences at the surface structure level between the WT and the *elr* operon-overexpressing strain (data not shown). This indicated that no major structural modification was detected under the tested conditions. Since high expression levels of surface proteins can modify physicochemical properties of bacterial cell surface such as charge or hydrophobicity [19, 20], we compared the affinity of bacterial cells of WT and P^+^-*elrA-E* strains to the solvents using a MATS test as described by Bellon-Fontaine et al. [21]. Both strains exhibited similar affinity for the apolar solvents decane (~40 %) and hexadecane (~30 %) and for the acidic solvent chloroform (~70 %), indicating no major changes of the surface hydrophobicity upon *elr* overexpression. In turn, the affinity of the strain P^+^-*elrA-E* for the basic solvent ethyl acetate (~35 %) increased significantly compared to the WT (<1 %), indicating that expression of *elr* operon enhances the negative charge of the bacterial cells. Thus we hypothesize that poor adhesion of strain P^+^-*elrA-E* may result from repulsive forces between the negatively charged macrophage membrane and bacterial surface, which is loaded with Elr proteins.

### Overexpression of *elr* operon increases *E. faecalis* virulence

**Fig. 4:**
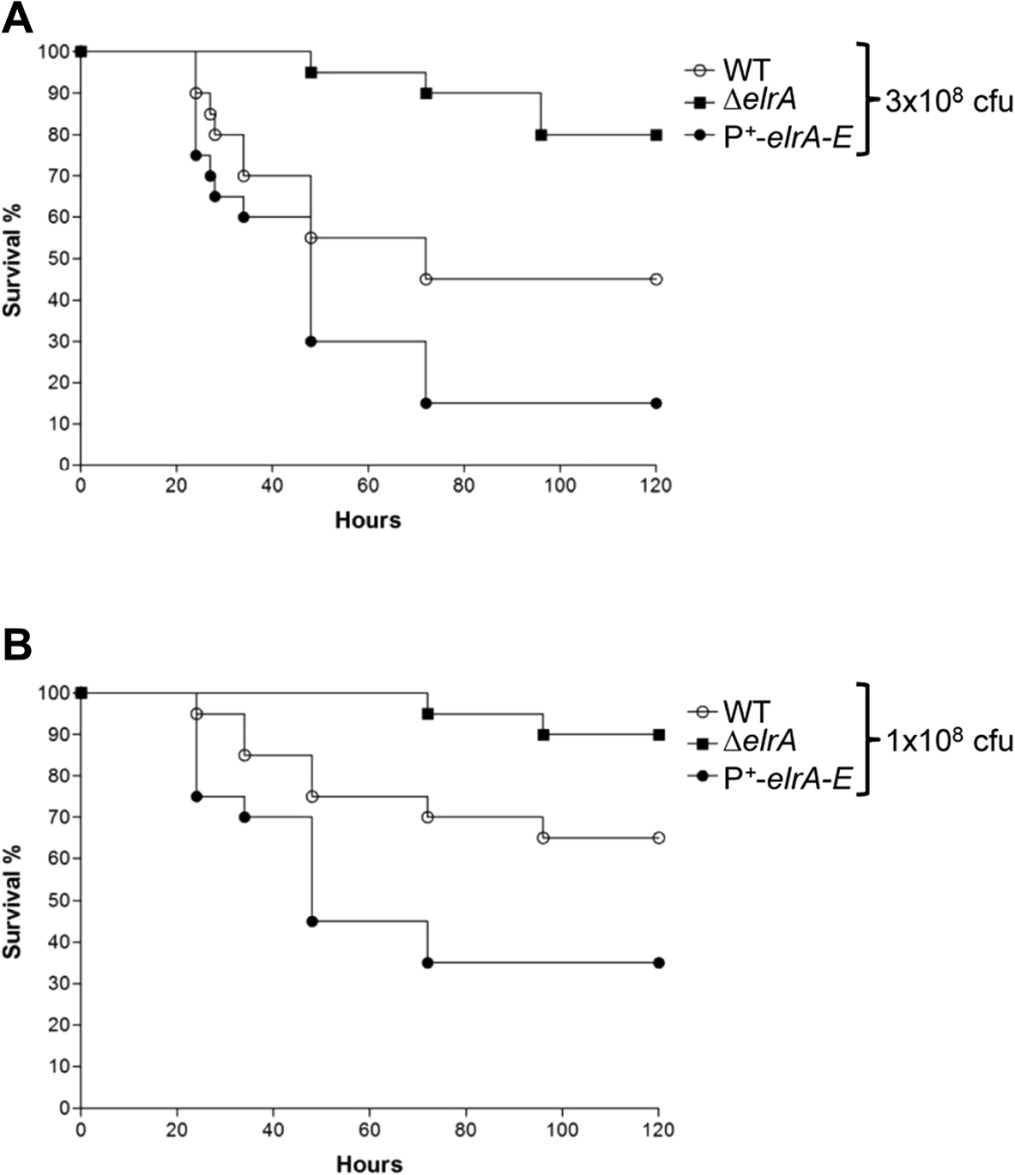
Effect of overexpression of *elr* operon on *E. faecalis* virulence. Kaplan-Meier survival analysis in a mouse peritonitis model with the *E. faecalis* WT strain (open circles), the Δ*elrA* strain (squares), and the P^+^-*elrA-E* strain (closed circles). A total of 10 mice were infected intraperitoneally with ~3 x 10^8^ (**a**) or ~1 x 10^8^ (**b**) CFU of each strain. For pairwise comparisons of P^+^-*elrA-E* / WT and P^+^-*elrA-E* / WT, *P* values were < 0.05 for each inoculum

**Fig. 5:**
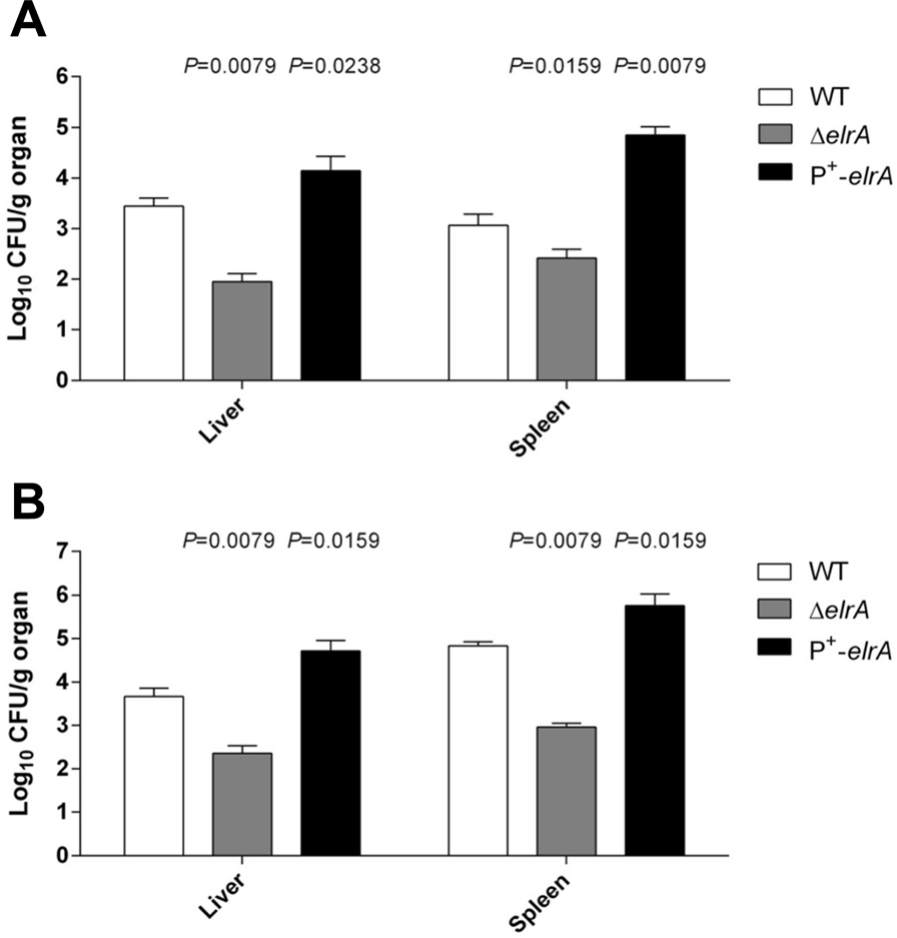
Overexpression of *elr* operon in *E. faecalis* increases bacterial dissemination in mice. *E. faecalis* organ burden in 10 mice were infected intraperitoneally with ~3 x 10^8^ (**a**) or ~1 x 10^8^ (**b**) CFU of each strain. The results represent the means and standard deviations of the number of bacteria able to colonize the spleen and liver at 24 h postinfection

Inactivation of ElrA reduces virulence in a mouse model of peritonitis [4] and we show that overexpression of *elr* operon impairs phagocytosis *in vitro*. We hypothesized that overexpression of *elr* may enhance dissemination, and thus *E. faecalis* virulence. To test this hypothesis, we assessed the survival of mice following peritoneal infection with WT, P^+^-*elrA-E*, or Δ*elrA* strains. Mice were injected with three different doses of WT or mutant strains, and the mortality rates were compared. No differences in mortality levels were found when mice were infected with 10^9^ CFU of WT or mutant strains (data not shown). Interestingly, mortality was significantly increased for mice infected with 3 x 10^8^ and 1 x 10^8^ CFU of P^+^-*elrA-E* strain compared to WT and Δ*elrA* strains at 72 h post-infection (Fig. 4). Eighty five percent of the mice infected with 3 x 10^8^ CFU of P^+^-*elrA-E* strain died, whereas 45 % and 10 % mice died when infected with the WT and Δ*elrA* strains, respectively (*P* = 0.049 and *P* < 0.0001) (Fig. 4A). Similarly, 65, 30, and 5 % of the mice infected with 1 x 10^8^ CFU of P^+^-*elrA-E*, WT, and Δ*elrA* strains died, respectively (*P* = 0.044 and *P* < 0.0001) (Fig. 4B). These results show that overexpression of *elr* operon increases *E. faecalis* virulence. We also compared the dissemination of the WT, P^+^-*elrA-E*, or Δ*elrA* strains in organs of mice at 24 h postinfection by determining bacterial loads (Fig. 5A). A 0.70- and 1.79-log10 increase in the bacterial counts in the liver and spleen, respectively, were observed for the P^+^-*elrA-E* compared to the WT and Δ*elrA* strains, respectively, when mice were challenged with inocula of 1x10^8^ CFU. Similar trends were observed with inocula of 3x10^8^ CFU (1.05- and 0.93-log10, respectively), although to a somewhat lesser extent (Fig. 5B). These results indicate that the virulence phenotype correlates with higher dissemination of the strain P^+^-*elrA-E*. The correlation between increased virulence and avoidance of phagocytosis observed *in vitro* corroborates our hypothesis that *elr* operon may be involved in the evasion of the immune response by *E. faecalis*. We previously linked the attenuated virulence of an *elrA* deficient strain with the decreased organ burden and survival in peritoneal macrophages using an *in vivo–in vitro* infection model [4]. These new data suggest that expression of *elrA* and/or *elr* operon contributes to the escape of *E. faecalis* from phagocytosis *in vivo*, promoting dissemination and enhancing virulence of the pathogen.

## Discussion

Previous studies have shown that *E. faecalis* survives into peritoneal macrophages better than non-pathogenic bacteria [8]. Since then, *E. faecalis* virulence factors able to interfere with uptake and survival in macrophages have been described [22]. We previously linked the attenuated virulence of an *E. faecalis* strain deleted for *elrA* with decreased organ burden and survival in peritoneal macrophages [4]. In this study, we show that overexpression of *elr* operon by *E. faecalis* confers resistance to phagocytosis by interfering with bacterial adhesion to macrophages. We also correlated *E. faecalis* avoidance of phagocytosis observed *in vitro* with increased virulence and dissemination in a mouse peritonitis model. These data contrast with our previous report that WT and Δ*elrA* strains were evenly phagocytosed [4]. Nevertheless, these studies are difficult to compare since macrophage infections were performed differently (i.e. *in vivo versus in vitro* infection and duration of infection). Moreover, the expression level of Elr proteins *in vivo* is unknown. The tight control of expression of the *elr* operon suggests that the operon may be required in specific conditions that remain to be identified [4, 5]. The 162-fold increased level of *erlA* transcript in *E. faecalis* strain MMH594 grown in urine [6], supports that expression of Elr proteins may vary in response to host-derived cues. We assume that *elr* operon may enhance *E. faecalis* virulence by promoting initial dissemination in the host after escape of bacteria from phagocytosis, but also by contributing to *E. faecalis* survival within infected macrophages depending on the tissues or cell types encountered by *E. faecalis*. From this study we propose that high-level expression of *elr* operon may, in some circumstances, occur *in vivo* and promotes escape of *E. faecalis* from phagocytosis.

The present study also revealed that ElrA requires at least one other *elr* gene to be expressed at a detectable level and confirmed that *elrA* gene is cotranscribed with the other *elr* genes [4]. The *elr* operon is a typical gene cluster of WxL surface proteins that associate non-covalently to the peptidoglycan of low-GC gram-positive bacteria. The operonic organization of the *elr* operon and the need of at least one other protein encoded by *elr* operon for ElrA production *in vitro* further support the hypothesis that cell-surface proteins, encoded by the *elr* operon, may participate in the formation of a multicomponent complex at the surface as it has been previously proposed [1, 2, 4]. Based on recent work by Galloway-Pena *et al.* who showed that WxL and DUF916 proteins interact *in vitro* [3], we believe that ElrA may be protected from degradation by interacting with at least another *elr*-encoded protein. If neither surface appendages nor modification could be observed upon overexpression of *elr* operon, other experiments are needed to establish if *elr* operon drives the formation of a surface complex in *E. faecalis*. Nevertheless, overexpression of Elr proteins seems to increase the negative charge of the bacterial surface, suggesting that *E. faecalis* evasion of phagocytosis by immune cells is driven by electrostatic repulsion. Even if *elr* overexpression emphasizes the steric or charge hindrance by Elr proteins *in vitro*, one cannot exclude that similar physicochemical changes occur *in vivo* in response to environmental cues [6], and confers to the *E. faecalis* cells anti-adhesion properties that promote escape from phagocytosis. These *in vitro* findings are reminiscent of the acidic LRR protein Slr from *Streptococcus pyogenes* that is involved in phagocytosis evasion [23], probably by enhancing the anti-adhesive properties of streptococcal cells. Another possibility would be that high level of Elr proteins sterically hinders *E. faecalis*-associated molecular patterns important for recognition by scavenger receptors. Altogether, this work shows that expression of *elr* operon contributes to the escape of *E. faecalis* from phagocytosis, promoting dissemination and enhancing virulence of the pathogen. Further investigations will focus on characterizing the precise role of each of the Elr proteins.

## Conclusions

In summary, this work shows that high-level expression of *elr* operon by *E. faecalis* increases virulence and confers resistance to phagocytosis, probably through charge repulsion. Consistently, the strain expressing *elr* operon displays stabilization of ElrA, further supporting that Elr proteins form an extracellular protein complex as part of the virulence process. Structural and functional characterization of the Elr proteins will help to understand *E. faecalis* pathogenesis and provide clues on WxL- and associated proteins of low-GC Gram-positive bacteria.

## Methods

### Reagents

All reagents were obtained from Sigma-Aldrich (St. Louis, MO), unless otherwise stated.

### Bacterial strains and plasmids

Bacterial strains and plasmids used in this work are listed in Table 1. *E. faecalis* strains were grown in M17 medium supplemented with 0.5 % glucose (GM17) at 37 °C without aeration. *Escherichia coli* strains were grown aerobically in Luria-Bertani medium at 37 °C. Plasmid constructions were first established in *E. coli* TG1 strain and then transferred into *E. faecalis* by electrotransformation using a Bio-Rad Gene Pulser Electroporator (Bio-Rad Laboratories). *E. faecalis* strains expressing green-fluorescent protein (GFP) were obtained by electroporation with pMV158-GFP plasmid [24]. Recombinant bacteria were selected by the addition of antibiotics as follows: for *E. faecalis* chloramphenicol 4 μg/ml, tetracycline 4 μg/ml and erythromycin (Ery) 30 μg/ml; for *E. coli*, chloramphenicol 10 μg/ml and ampicillin 100 μg/ml. DNA manipulations were performed as previously described [25].

**Table 1:** Strains and plasmids used in this work

Strain	Designation relevant characteristics	Source or Reference
*E. faecalis*		
WT	Fus^r^ Rif^r^; plasmid-free wild-type strain	[35]
Δ*elrA*	OG1RF Δ*elrA*	[4]
P^+^-*elrA-E*	OG1RF P_*aphA3*_::*elrA-E*	This work
P^+^-Δ*elrA*	OG1RF P_*aphA3*_::Δ*elrA*	This work
P^+^-*elrA-*Δ*elrB-E*	OG1RF P_*aphA3*_::*elrA*-Δ*elrB-E*	This work
P^+^^-Δ*elrA-E*^	OG1RF P_*aphA3*_::Δ*elrA-E*	This work
*E. coli*		
TG1	*supE hsdD5 thi* (Δ*lac-proAB*) F’ (*traD36 proAB-lacZ*Δ*M15*)	[36]
BL21(λDE3)	F^−^ *ompT gal dcm hsdSB*(rB^−^mB^−^) λDE3	[37]
Plasmids		
pACYC177	Amp^r^, Kan^r^, ori p15A	[38]
pET2817	Amp^r^, ori colE1, T7 promoter, His-Tag coding sequence	[39]
pGEM-T easy	Amp^r^, ori ColE1, linearized with 3’ T overhangs	Promega
pGhost9	Erm^r^, ori pWV01, *repA*(Ts)	[27]
pMV158-GFP	pMV158 with the gene encoding the green fluorescent protein	[24]
pTCV-*lac*(P_*aphA3*_)	Tet^r^, ori ColE1, ori pAMβ1, *lacZ* harboring P_*aphA3*_ promoter	[16]
pVE14009	Erm^r^, ori pWV01, *repA*(Ts), with *elrA* deletion	[4]
pVE14047	Amp^r^, pET2817 with 6x His::ElrA	This work
pVE14142	Amp^r^, Kan^r^, ori p15A, ‘*elrR*-*elrA*’ region	This work
pVE14145	Amp^r^, Kan^r^, ori p15A, ‘*elrR*-P_*aphA3*_::*elrA*’ region	This work
pVE14146	Erm^r^, ori pWV01, *repA*(Ts), ‘*elrR*-P_*aphA3*_::*elrA*’ region	This work
pVE14178	Amp^r^, ori colE1, with *elrA-E* deletion	This work
pVE14179	Amp^r^, ori colE1, with *elrB-E* deletion	This work
pVE14450	Erm^r^, ori pWV01, *repA*(Ts), with *elrB-E* deletion	This work
pVE14455	Amp^r^, Kan^r^, ori p15A, with P_*aphA3*_ *::elrA-E* deletion	This work
pVE14456	Erm^r^, ori pWV01, *repA*(Ts), with P_*aphA3*_ *::elrA-E* deletion	This work
pVE14457	Erm^r^, ori pWV01, *repA*(Ts), with P_*aphA3*_::*elrA* deletion	This work

### Cell line and culture conditions

The RAW 264.7 mouse macrophage cell line (ATCC®−TIB-71) was maintained in DMEM supplemented with 10 % heat-inactivated fetal bovine serum (FBS) and 2 mM l-glutamine [26]. For phagocytosis assays, cells were seeded at 0.5 × 10^6^/well into 12-well tissue culture plates (TPP, Domique Dutscher, Brumath, France) and incubated overnight at 37 °C under 6 % CO2. For microscopy experiments, cells were cultured in tissue culture plates containing poly-L-lysine pretreated coverslips for microscopy or on Lab-tek chamber slides (Nunc, Domique Dutscher). Comparative analysis of phagocytosis using either heat-inactivated serum or serum-free media (Macrophage-SFM, GIBCO, Invitrogen) did not show differences (data not shown). Thus, for practical reasons we decided to use heat-inactivated serum in all experiments of this work.

### Generation of anti-ElrA rat polyclonal antibodies

Recombinant ElrA was purified to produce polyclonal rat anti-ElrA antibodies by Proteogenix (Oberhausbergen, France). Briefly, a DNA fragment encoding *elrA* was PCR-amplified from *E. faecalis* chromosomal DNA using OEF275 and OEF276 primers (Table 2). The PCR product was digested with *Bam*HI/*Pci*I and cloned into purified *Bam*HI/*Nco*I-digested pET2817 vector backbone, resulting in plasmid VE14047 which was transformed into *E. coli* BL21(λDE3). For ElrA production and purification, the resulting recombinant strain was cultured at 25 °C and induced with 1 mM of IPTG (isopropyl β-D-1-thiogalactopyranoside) for 5 hrs. Recombinant 6xHis::ElrA protein was purified under denaturing conditions on Ni-NTA columns using the QIAexpress kit (Qiagen, Courtaboeuf, France).

**Table 2:** Primers used in this study

Name	Sequence 5'-3'	Source or Reference
OEF9	TTGACCATCACGAGATACC	This work
OEF13	CTATCTTGGTCAAAAGAGCG	This work
OEF15	TATTCGATGTTGGCGTTGG	[4]
OEF18	GGAGGATGCGATTGTTTCG	[4]
OEF212	CTCTTCTGCCGATGAAGTTTCTGG	[4]
OEF275	CAAACATGTTAGAAACGACCGAAACAATCGC	This work
OEF276	TTGGATCCACTCACCCCCTATTTTGC	This work
OEF343	GCGAATTCGAAGATCTGAGAAAATATCAGGAGGTGAAG	This work
OEF344	ATGGATCCAGACGGAGTAGGTTATTTGC	This work
OEF345	TTCTCAGATCTTCGAATTCGCTGAATATCAACTGAAAATGGG	This work
OEF346	ATCTCGAGTTGCGTATTTCGGATTTAGCC	This work
OEF49	CACGCTGTACGATCAGCAAC	This work
OEF595	CAATCCTAATAGCAATACACC	This work
OEF596	GGTGTATTGCTATTAGGATTGTGCCTGTTCATCATTTTACG	This work
OEF598	CGAAACAATCGCATCCTCCTGCCTGTTCATCATTTTACG	This work
Vlac1	GTTGAATAACACTTATTCCTATC	[16]
Vlac2	CTTCCACAGTAGTTCACCACC	[16]

### Construction of mutant and over-expressing strains of *E. faecalis*

The *E. faecalis elrA* gene is part of a five-gene operon *elrA* (OG1RF_12055), *elrB* (OG1RF_12054), *elrC* (OG1RF_12053), *elrD* (OG1RF_12052), and *elrE* (OG1RF_12051) (Fig. 1), encoding putative surface proteins of unknown function. To circumvent the lack of ElrA production *in vitro*, we constructed a genetically modified *E. faecalis* strain harboring the constitutive promoter P*aphA3* (hereafter named P^+^), instead of the native promoter, P*elrA*, upstream of the whole *elr* operon (i.e., *elrA-E*, Fig. 1). This genetically modified strain (called P^+^-*elrA*-*E*), was constructed by a double cross-over event using the pGhost9 plasmid [27]. Briefly, two overlapping fragments were PCR-amplified from *E. faecalis* OG1RF chromosomal DNA with primers OEF343/OEF344 and OEF345/OEF346 (Table 2). The two PCR products were then fused by PCR using the external primers OEF344/OEF346, and the resulting product was cloned into purified *Xho*I-*Bam*HI-digested pACYC177 vector, resulting in plasmid pVE14142. The P*aphA3* promoter was PCR-amplified with primers Vlac1 and Vlac2 from pTCV-*lac*(P*aphA3*) plasmid [16]. An *Eco*RI-*Bam*HI fragment, containing the P*aphA3* promoter, was then cloned into *Eco*RI-*Bgl*II-digested pVE14142 vector to obtain plasmid pVE14145. Then, a 2.3 kb *Xho*I-*Eae*I fragment from pVE14145 plasmid (containing the promoter and the targeted region) was cloned into pGhost9 vector to generate the final vector pVE14146. This plasmid was established in *E. faecalis* OG1RF strain and a markerless insertion of P*aphA3* upstream of the *elrA-E* operon was performed as previously described [1]. Correct integration of P*aphA3* into the chromosomal locus was confirmed by sequencing. All the following mutant constructs were performed using P^+^−*elrA-E* strain as a recipient in order to have the same genetic background (Table 1). For the construction of a strain expressing only *elrA* under the control of P*aphA3* promoter, a fused DNA fragment using primers OEF13/OEF595 and OEF596/OEF49 amplified from OG1RF strain DNA was cloned into pGEM-T easy vector (Promega) to generate pVE14179. A 4.5 kb *Pst*I fragment was then cloned into *Pst*I-digested pGhost9 to obtain plasmid pVE14450 and established in P^+^-*elrA-E* strain to obtain the P^+^-*elrA-*Δ*elrB-E* strain. For the construction of a strain expressing *elr* operon lacking *elrA*, a 6.6 kb *Bst*/*Ape*I fragment from pVE14009 was cloned into *Bst*/*Ape*I-digested pVE14146 vector, resulting in pVE14457. This plasmid was established in P^+^-*elrA*-*E* strain to obtain the P^+^-Δ*elrA* strain. To inactivate the whole *elr* operon (i.e., *elrA*-*E*), we first generated an in-frame deletion of the whole operon by PCR. For this, we used OEF15/OEF18 and OEF598/OEF49 primers described for the first PCR. The two PCR products were fused by PCR using external primers OEF49/OEF15, and the resulting product was cloned into pGEM-T, resulting in plasmid pVE14178. A *BstA*PI/*Aat*II 890bp DNA fragment from pVE14178 was then cloned into pVE14145 to generate pVE14455. The final plasmid was generated by cloning a 4.5 kb *Xho*I DNA fragment from pVE14455 vector into *Xho*I-digested pGhost9 to obtain pVE14456. This plasmid was established in P^+^-*elr*A-E strain and the resulting strain was named P^+^-Δ*elrA-E*. All expected modifications or deletions were confirmed by sequencing.

### Preparation of protein extracts, SDS gel electrophoresis, and immunoblot analysis

Total protein extraction from bacteria, SDS-PAGE, and Western blot immunodetection were carried out using standard methods (24) with some modifications. Strains were grown at 37 °C overnight and then diluted 100-fold and grown under the same conditions to an OD_600_~1. Protein crude extract was obtained by trichloroacetic acid (TCA) precipitation by mixing 800 μl of bacterial culture with 200 μl of ice cold TCA solution (100 % w/v). The protein pellet was then obtained by centrifugation and recovered directly into SDS sample buffer. Anti-ElrA antibody was used at a dilution of 1:500 for Western blot immunodetection.

### RNA isolation and Northern blotting

Total RNA was extracted as previously described [28]. Northern blots were performed on 40 μg of total RNA separated on a 0.9 % denaturing agarose gel as previously described [29]. Specific oligonucleotides OEF9 and OEF212 were used to detect *elrA* transcripts. Oligonucleotides were labelled with [γ-32P]-ATP and T4 polynucleotide kinase (NEB Biolabs) according to the recommendations of the manufacturer (NEB Biolabs). Analysis was performed from RNA extracted from two independent experiments.

### Phagocytosis assay with RAW macrophages

Fluorescent *E. faecalis* were grown on GM17 plates containing erythromycin (GM17-Ery), with a single colony subsequently being selected and grown overnight in GM17-Ery broth. A 100 μl aliquot was then transferred into 10 ml of fresh GM17-Ery and incubated until cultures reached an OD_600_ ~1. Bacteria were then pelleted by centrifugation, washed three times with PBS, and adjusted to a concentration of 1 × 10^9^ CFU/ml in supplemented DMEM. The number of bacteria present in each suspension was confirmed by plating onto solid GM17-Ery.

For phagocytosis experiments, adherent RAW cells were infected with fluorescent *E. faecalis* at a multiplicity of infection (MOI) of 100:1 (bacterium/cell ratio). After 30 min of interaction, cells were washed twice with PBS, recovered with cell dissociation buffer (GIBCO, Invitrogen), washed again, and finally fixed in 3 % paraformaldehyde (PFA) solution. Fluorescence of RAW cells due to infecting bacteria was detected by a flow cytometer in the FL-1 channel. The phagocytosis index (PI) was calculated using the percent of fluorescent macrophages after *E. faecalis* wild-type (WT) strain infection and applying the following formula: PI = (percent of fluorescent macrophages after infection X 100 /percent of fluorescent macrophages after WT infection) [30, 31]. Results are expressed as the mean ± SEM from three independent experiments usually performed in duplicate or triplicate.

### Bacterial adhesion assay

To separate adhesion from subsequent steps of phagocytosis, cells were pretreated 30 min with 1 μg/ml of cytochalasin D (CytD), an actin polymerization inhibitor, as described [17]. A CytD (1000X) stock solution in DMSO was prepared according to manufacturer's recommendations and stored at −20 °C. DMEM supplemented medium (see above) was used to dilute stock solution. RAW cells were seeded at 1 × 10^6^/well into 6-well tissue culture plates (TPP, Dominique Dutscher) and incubated O/N at 37 °C under 6 % CO2. Macrophages pre-treated with CytD were first washed twice with fresh medium and then infected at a MOI of 100:1, similar to phagocytosis analysis above; CytD-untreated and uninfected macrophages were used as negative controls. Fluorescence in RAW cells due to infecting bacteria was detected by flow cytometry. Adhesion Index (AI) = (percent of GFP^+^ macrophages pre-treated with CytD, after infection by the *E. faecalis* mutant strain X 100/percent of GFP^+^ macrophages pre-treated with CytD after WT infection).

### Fluorescence and electron microscopy

Raw macrophages were seeded in 12-well cell culture plates on a glass slide and infected with GFP-labeled *E. faecalis* wild-type (WT) or P^+^-*elrA-E* strains at a MOI of 1:100, with uninfected macrophages serving as negative control. After 30 min of interaction, macrophages were washed twice with PBS, fixated and immunolabeled with *Streptococcus* group D antiserum (BD Diagnostics, Le Pont de Claix, France) as previously described [4]. Fluorescence was examined using a Carl Zeiss microscope (Axiovert 200 M, in the ApoTome mode) at MIMA2 platform (INRA, Jouy en Josas). Images were processed with Axiovision version 4.6 (Carl Zeiss).

Imaging of bacterial-cells interaction was performed using a Hitachi S-4500 scanning electron microscope (SEM) at the MIMA2 imaging platform. Macrophages were seeded in 12-well cell culture plates and infected with either *E. faecalis* wild-type (WT) or P^+^-*elrA-E* strains at a MOI of 1:100, with uninfected macrophages serving as negative control. After 30 min of interaction, macrophages were washed twice with PBS, recovered with cell dissociation buffer (GIBCO, Invitrogen), washed again, and suspended in a fixative solution and treated as previously described [32].

Preparation of bacterial samples for transmission and scanning electron microscopy was performed as previously described [32, 33]. Thin-sections and negative-stains were observed with a Zeiss EM902 electron microscope operated at 80 kV (MIMA2 - UR 1196 Génomique et Physiologie de la Lactation, INRA, plateau de Microscopie Electronique, 78352 Jouy-en-Josas, France). Microphotographies were acquired using MegaView III CCD camera and analyzed with the ITEM software (Eloise SARL, Roissy CDG, France).

### Microbial adhesion to solvents

Microbial adhesion to solvents (MATS) analysis was carried out as described previously by Bellon-Fontaine and collaborators [21]. In brief, a single colony of each of the *E. faecalis* strains studied was subcultured four times in BHI and harvested at stationary phase. Bacterial cells were centrifuged at 5000 ×g for 8 min and washed twice in 0.15 M NaCl and re-suspended to a final OD_400_ ~0.8. Bacterial suspensions (2.4 ml) were vortexed for 1 min with 0.4 ml of highest purity grade chloroform (Sigma-Aldrich), hexadecane (Sigma-Aldrich), ethyl acetate (Merck), or decane (Merck). The emulsion was left to stand for 20 min to allow complete phase separation, and the OD_400_ of 1 ml from the aqueous phase was measured. Affinity of the cells for each solvent (% affinity) = ((OD_f_-OD_i_)/ OD_i_)x100 where OD_*i*_ is the initial optical density of the bacterial suspension before mixing with the solvent, and OD_*f*_ the final absorbance after mixing and phase separation. Analysis was performed twice in triplicate.

### Mouse peritonitis model

The mouse experiments were approved by the Institutional Animal Use and Care Committee at the Università Cattolica del Sacro Cuore, Rome, Italy (permit number Z21, 1 November 2010), and authorized by the Italian Ministry of Health, according to the Legislative Decree 116/92, which implemented the European Directive 86/609/EEC on laboratory animal protection in Italy. Animal welfare was routinely checked by veterinarians of the Service for Animal Welfare.

Virulence of strains OG1RF, Δ*elrA*, and P^+^-*elrA-E* was tested as described previously [4]. The inoculum size was confirmed by determining the number of CFU on brain heart infusion agar. Each inoculum was 10-fold diluted in 25 % sterile rat fecal extract prepared from a single batch as previously described [34]. Groups of 10 ICR outbred mice (Harlan Italy Srl, San Pietro al Natisone, Italy) were challenged intraperitoneally with 1 ml of each bacterial inoculum, housed five per cage, and fed *ad libitum*. A control group of mice was injected with 25 % sterile rat fecal extract only. Survival was monitored every 3 to 6 h. In another set of experiments, groups of mice were killed 24 h postinfection, and livers and spleens were removed, weighed, homogenized, and serially diluted in saline solution for colony counts.

### Statistical analysis

Statistics were performed using GraphPad Prism (Version 4.00 for Windows, GraphPad Software, San Diego California, USA). One-way analysis of variance (ANOVA) was followed by Dunnett's multiple-comparison test when comparing multiple groups for one factor. For animal experiments, survival estimates were constructed by the Kaplan-Meier method and compared by log rank analysis, and comparisons with *P* values of <0.05 were considered to be significant.

## Additional file

Additional file 1: Figure S1. Bacterial adhesion assay. RAW macrophages were pretreated with cytochalasin D (CytD) or not (-) before infection with *E. faecalis* strains WT and P^+^-*elrA-E* expressing GFP. After 30 min of interaction, cells were washed twice with PBS, recovered with cell dissociation buffer. GFP positives macrophages were detected by flow cytometry. Graphs represent green fluorescence intensity. Results are representative of two independent experiments. In the absence of CytD pretreatment, the population of GFP-labeled macrophages infected with the P^+^-elrA-E strain (55.5 %) is decreased compared to the macrophages infected with the WT strain (86.6 %). CytD is known to inhibit actin rearrangement and blocks bacterial entry. To establish whether differential GFPlabeling of macrophages resulted from adhesion or entry defect of strain P^+^-elrA-E, similar experiment was performed on macrophages pre-treated with CytD. When infected with the WT strain, the population of GFP-labeled pre-treated macrophages (77.9 %) slightly decreased compared to untreated ones. This observation indicates that the majority of the GFP-labeled macrophages detected harbor adherent GFP-bacteria at their surface. In contrast, the intensity and the population of GFP-labeled pre-treated macrophages infected with the P^+^-elrA-E strain (28.4 %) decreased drastically compared to untreated ones, indicating that P^+^-elrA-E bacteria adhered less efficiently to macrophages.
