# Supplementary material for: Overexpression of Enterococcus faecalis elr operon protects from phagocytosis
Source: BMC Microbiol. 2015 May 25;15:112. doi: 10.1186/s12866-015-0448-y (PMC4522977; doi:10.1186/s12866-015-0448-y)
Supplement: Additional file 1: Figure S1. — Bacterial adhesion assay. RAW macrophages were pretreated with cytochalasin D (CytD) or not (-) before infection with E. faecalis strains WT and P+-elrA-E expressing GFP. After 30 min of interaction, cells were washed twice with PBS, recovered with cell dissociation buffer. GFP positives macrophages were detected by flow cytometry. Graphs represent green fluorescence intensity. Results are representative of two independent experiments. In the absence of CytD pretreatment, the population of GFP-labeled macrophages infected with the P+-elrA-E strain (55.5 %) is decreased compared to the macrophages infected with the WT strain (86.6 %). CytD is known to inhibit actin rearrangement and blocks bacterial entry. To establish whether differential GFPlabeling of macrophages resulted from adhesion or entry defect of strain P+-elrA-E, similar experiment was performed on macrophages pre-treated with CytD. When infected with the WT strain, the population of GFP-labeled pre-treated macrophages (77.9 %) slightly decreased compared to untreated ones. This observation indicates that the majority of the GFP-labeled macrophages detected harbor adherent GFP-bacteria at their surface. In contrast, the intensity and the population of GFP-labeled pre-treated macrophages infected with the P+-elrA-E strain (28.4 %) decreased drastically compared to untreated ones, indicating that P+-elrA-E bacteria adhered less efficiently to macrophages. [file 12866_2015_448_MOESM1_ESM.pdf]

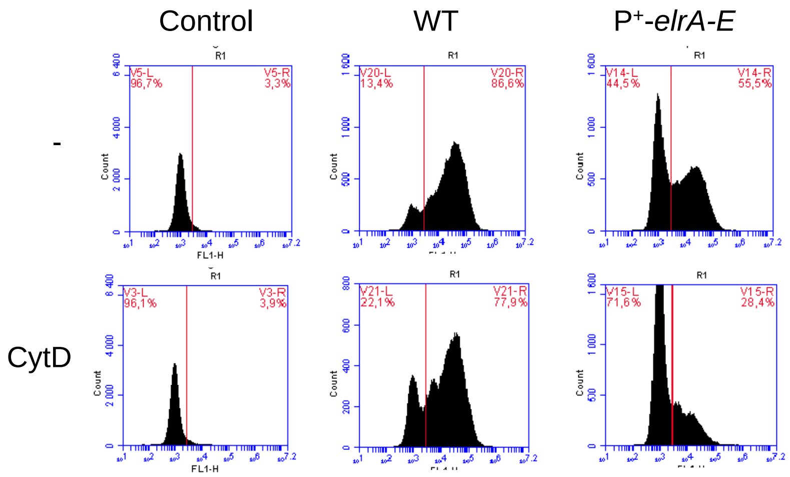

FIG. S1. Bacterial adhesion assay. RAW macrophages were pretreated with cytochalasin D (CytD) or not (-) before infection with *E. faecalis* strains WT and P^{+}-*elrA-E* expressing GFP. After 30 min of interaction, cells were washed twice with PBS, recovered with cell dissociation buffer. GFP positives macrophages were detected by flow cytometry. Graphs represent green fluorescence intensity. Results are representative of two independent experiments.

In the absence of CytD pretreatment, the population of GFP-labeled macrophages infected with the P^{+}-elrA-E strain (55.5%) is decreased compared to the macrophages infected with the WT strain (86.6%). CytD is known to inhibit actin rearrangement and blocks bacterial entry. To establish whether differential GFP-labeling of macrophages resulted from adhesion or entry defect of strain P^{+}-elrA-E, similar experiment was performed on macrophages pre-treated with CytD. When infected with the WT strain, the population of GFP-labeled pre-treated macrophages (77.9%) slightly decreased compared to untreated ones. This observation indicates that the majority of the GFP-labeled macrophages detected harbor adherent GFP-bacteria at their surface. In contrast, the intensity and the population of GFP-labeled pre-treated macrophages infected with the P^{+}-elrA-E strain (28.4%) decreased drastically compared to untreated ones, indicating that P^{+}-elrA-E bacteria adhered less efficiently to macrophages.
